# Impact of mercury on photosynthetic performance of *Lemna minor*: a chlorophyll fluorescence analysis

**DOI:** 10.1038/s41598-023-39297-x

**Published:** 2023-07-27

**Authors:** Hanwant Singh, Deepak Kumar, Vineet Soni

**Affiliations:** Plant Bioenergetics and Biotechnology Laboratory, Department of Botany, Mohanlal Sukhadia University, Udaipur, Rajasthan 313001 India

**Keywords:** Biochemistry, Biological techniques, Biophysics, Plant sciences

## Abstract

The purpose of this study was to evaluate the effectiveness of chlorophyll fluorescence analysis in detecting the effects of mercury (Hg) treatment in duckweed species *Lemna minor*. The results showed that Hg treatment (ranging from 0.0 to 0.4 µM) significantly impacted the plant's photosynthetic ability, with a decrease in variable chlorophyll fluorescence, energy fluxes, density of reaction centers, and performance index. Complete inhibition of electron transport was observed in plants treated with high Hg concentrations, and the quantum yield of primary photochemistry and the ratio of dissipated energy to absorption both decreased with increasing Hg concentrations. Performance Index (PI) was significantly affected by the Hg concentrations, reaching zero in plants treated with the highest Hg concentration. Overall, JIP analysis was found to be an effective tool for detecting deleterious effects of Hg in plants.

## Introduction

The increasing environmental contamination of heavy metals (HMs), such as cadmium, mercury, lead, arsenic, zinc, copper, nickel, and chromium, has become a major global health and environmental issue in recent decades due to sustained industrialization and urbanization^1,2^. HMs are among the most prevalent contaminants and are of particular concern due to their persistence in the environment and inability to degrade over time^3,4^. The pollution of aquatic ecosystems, including the presence of micropollutants in treated wastewaters, drinking water, and surface or subsurface waters, is a significant problem worldwide and poses a potential risk to both the environment and human health^5^. The study of HM pollution is crucial in the field of plant stress physiology, as these metals can be toxic to plants and accumulate in food crops, thereby posing a risk to human health.

Naturally present in the earth’s crust, Hg is now facing a significant global pollution issue due to various anthropogenic activities such as mining and the combustion of fossil fuels. These activities release Hg into the air, which eventually settles into aquatic bodies or land and can be washed into water sources. Once deposited, certain microorganisms have the potential to transform it into methylmercury, that accumulates in fish, shellfish, and animals that consume fish^6^. In a study conducted by Boney in 1971, it was observed that exposure of the *Plumaria elegans* (red algae) to mercuric chloride resulted in significant growth inhibition. Specifically, it was found that concentrations of 1.0, 0.5, and 0.25 mg/l caused 50% growth inhibition after 6, 12, and 24 h, respectively^7^. De et al.^8^ conducted an experiment where floating water cabbage (*Pistia stratiotes*) was exposed to different concentrations of mercuric chloride for 2 days. The highest mercury dose resulted in reduced chlorophyll content, protein and RNA levels, dry weight, catalase and protease activity, while increasing the production of free amino acids^8^. Another study by Brown and Rattigan^9^ investigated the effects of mercuric chloride on Canadian pond weed (*Elodea canadensis*) and free-floating duckweed (*L. minor*) over 14 and 28 days. Concentrations of 7.4 mg/l and 1.0 mg/l in water caused 50% damage to the two plants, respectively. Additionally, the pond weed exposed to mercury showed 50% reduction in photosynthetic oxygen evolution in the dark and 90% in the light.

Chlorophyll (Chl) *a* fluorescence is a distinctive characteristic of all photosynthetic organisms, where light energy absorbed by Chl molecules is either used to drive photosynthesis or is dissipated as heat radiation or re-emitted as light photons^10^. Although fluorescence usually accounts for only 1–8% of total light absorbed, it provides valuable information about changes in the efficiency of photochemistry and heat scattering due to the interdependence of light-induced processes^11–13^. The measurement of Chl *a* fluorescence is convenient, non-invasive, highly sensitive, rapid, and reliable, and provides a quantitative probe of oxygenic photosynthesis, making it a widely used tool to determine the physiological status of plants^14–16^.

*Lemna minor*, also known as common duckweed, is widely distributed in a range of regions, from tropical to temperate and from freshwater to brackish waters^17^. The plant is native to Africa, Asia, Europe, and North America, but it also grows well in Australia and South America^18^. Researchers have found that *L. minor* has the ability to absorb high concentrations of HMs^19–22^. It has become a model system for ecotoxicological bioassays, genetic transformation, and industrial applications^23^. In addition to its applications in phytoremediation, *L. minor* has also been used to further our understanding of photoperiodic control of flowering^24^ and photosynthesis research^25^.

The objective of this study was to examine the effectiveness of using Chl fluorescence analysis to detect mercury-induced stress in plants, using the aquatic angiosperm *L. minor* as a test subject. Additionally, exploring the potential of *L. minor* as a biomonitoring tool for Hg contamination in aquatic environments could provide valuable insights into the extent and effects of Hg pollution.

## Results

### Biophysical studies of Hg tolerance in *L. minor*

The results of the study of Chl *a* fluorescence analysis showed that Hg treatment significantly impacted all photosynthetic parameters in *L. minor*. The presence of Hg in various concentrations caused a significant decrease in the plant's photosynthetic ability by reducing Chl fluorescence kinetics, energy fluxes, density of reaction centers, and performance indexes.

### Chl a fluorescence

**Figure 1:**
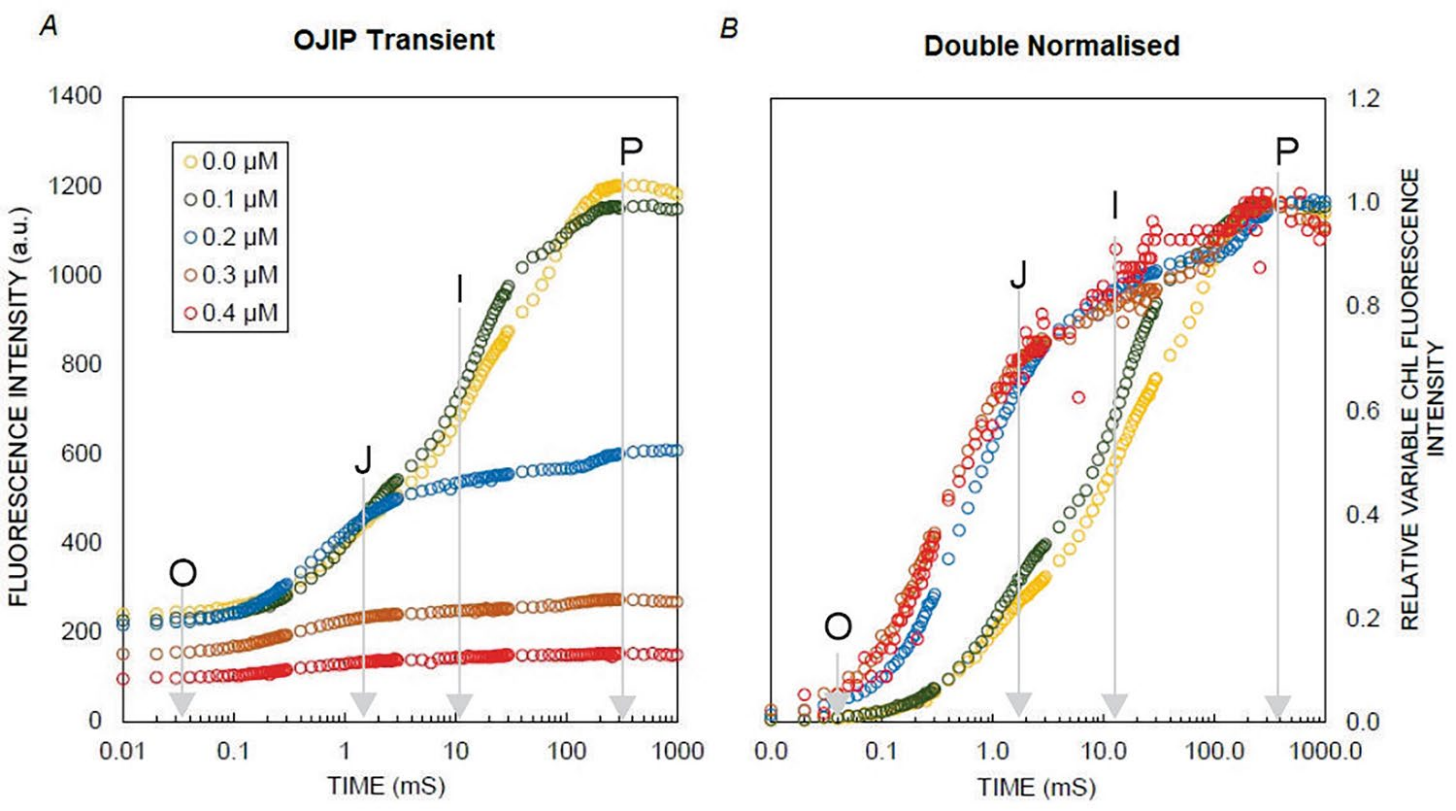
ChlF rises (**A**) and (**B**) Double normalized OJIP transient in *L. minor* plants exposed 48 h to different concentrations of HgCl_2_ (0.0–0.4 µM) and O, J, I, and P indicate PSII rapid fluorescence transients. Values presented are the average of three replicates.

The impact of Hg treatment was evident in the polyphasic Chl *a* fluorescence OJIP kinetics in *L. minor*. The Chl *a* fluorescence in plants grown under different Hg concentrations increased continuously from the minimum (F_O_) to the maximum (F_M_) fluorescence intensity. In control plants, two intermediate peaks, F_J_ and F_I_, were formed between F_O_ and F_M_, creating a typical OJIP curve (Fig. 1A and B). However, with increasing Hg concentration in the media, F_O_ (minimal fluorescence) decreased, with the lowest value recorded in plants treated with 0.4 µM. Similarly, F_M_ was significantly reduced as the concentration of Hg increased. When subjected to elevated Hg concentrations, the plants were unable to form a complete OJIP curve.

### Specific energy fluxes

**Figure 2:**
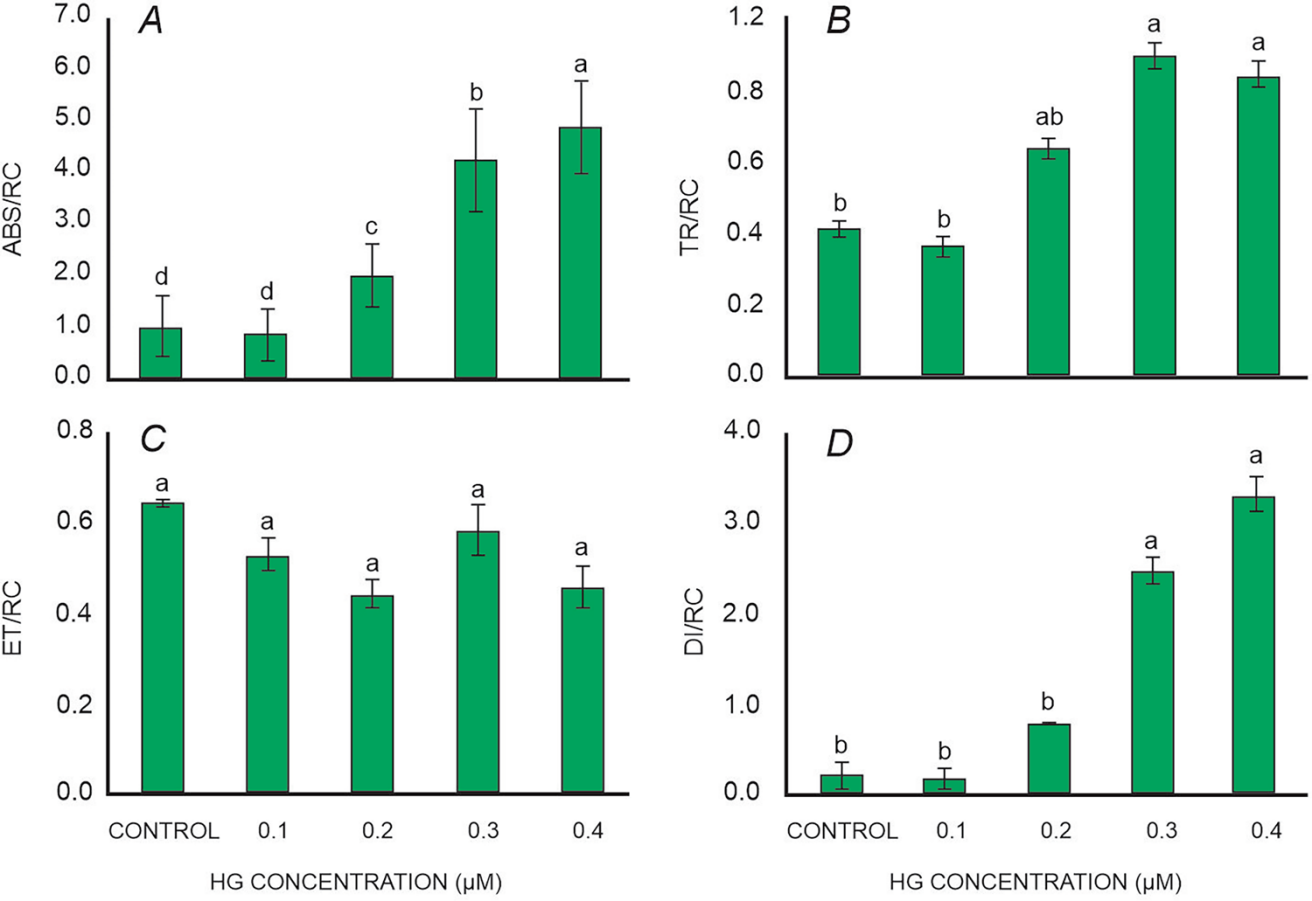
(**A**) Absorption per reaction center (ABS/RC), (**B**) trapped energy per reaction center (TR/RC), (**C**) electron transport per reaction center (ET/RC), (**D**) dissipation per reaction center (DI/RC) of *L. minor* exposed to various concentration of mercury. Values presented are the average of three replicates, and standard errors are represented by error bars. Different characters indicate significant differences among the results (p ≤ 0.05).

The specific energy fluxes [absorption flux per reaction center (ABS/RC), trapped energy flux per reaction center (TR/RC), electron transport flux per reaction center (ET/RC), and dissipated energy flux per reaction center (DI/RC)], which indicate the performance of active PSII reactions, underwent significant changes with increasing Hg concentrations in *L. minor*. At low Hg concentrations, ABS/RC did not change, but increased continuously with increasing Hg concentration in the media (Fig. 2A). The highest values of ABS/RC were observed in plants exposed to 0.4 µM Hg. Similarly, TR/RC increased continuously with increasing the concentration of Hg in *L. minor*, with the highest values recorded when the plants were exposed to 0.3 µM Hg for 48 h (Fig. 2B). The electron transport flux per reaction center (ET/RC) showed a slight decrease with increasing Hg treatment up to 0.3 µM, followed by a slight increase at higher concentrations in *L. minor* (Fig. 2C). Mild to moderate Hg treatment had a limited effect on the dissipated energy flux per reaction center (DI/RC). The results showed that DI/RC increased gradually at lower concentration of Hg treatment (0–0.2 µM) and then steadily increased until the higher concentration (0.4 µM) (Fig. 2D).

### Phenomenological energy fluxes

**Figure 3:**
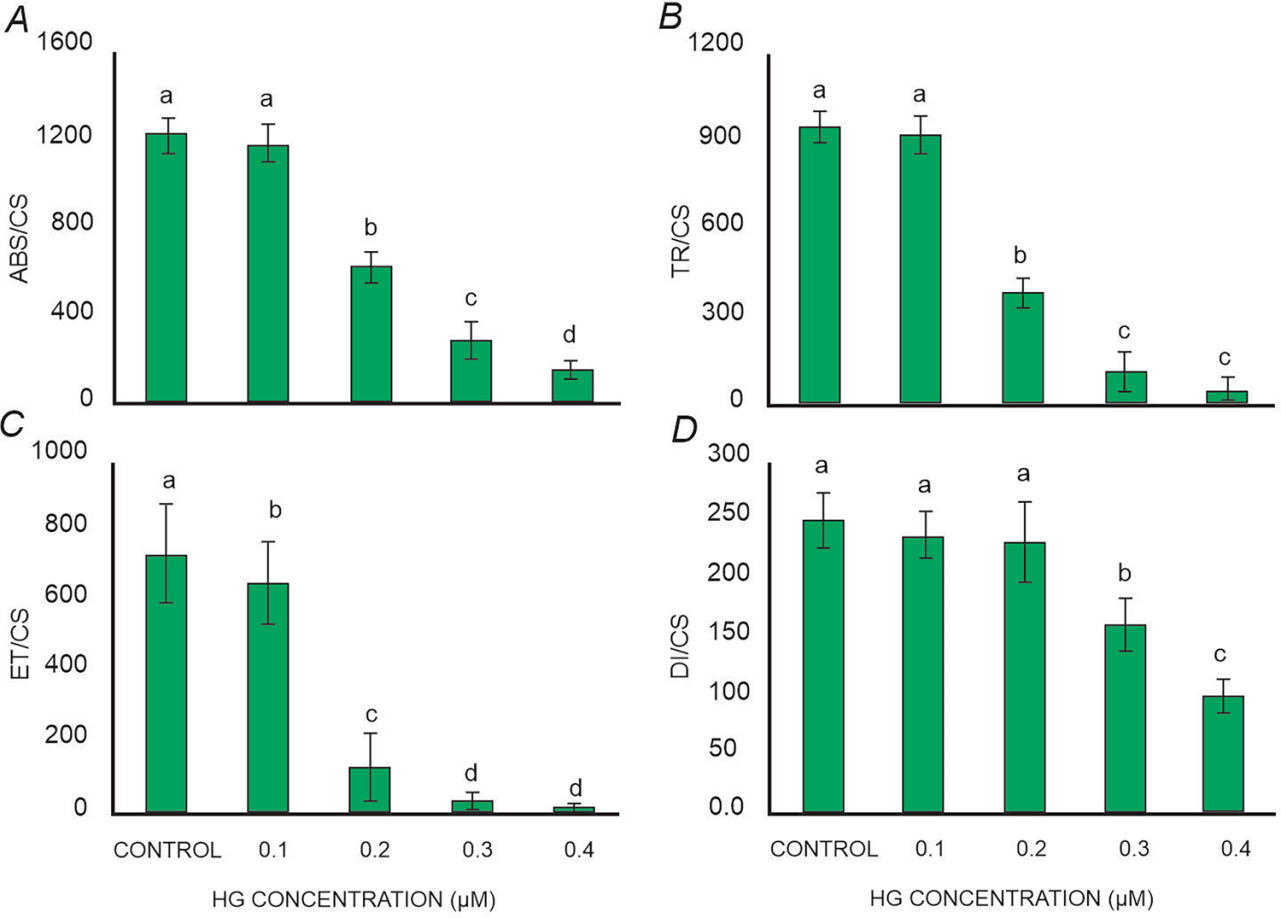
(**A**) Absorption per cross section (ABS/CS), (**B**) trapped energy per cross section (TR/CS), (**C**) electron transport per cross section (ET/CS), (**D**) dissipation per cross section (DI/CS) of *L. minor* espoused to various concentration of mercury. Values presented are the average of three replicates, and standard errors are represented by error bars. Different characters indicate significant differences among the results (p ≤ 0.05).

The results showed that Hg had a pronounced impact on the absorption flux per cross section (ABS/CS), trapped energy flux per cross section (TR/CS), electron transport flux per cross section (ET/CS), and dissipated energy flux per cross section (DI/CS) in *L. minor* exposed to different concentrations of Hg. ABS/CS remained relatively unchanged at 0.1 µM Hg concentration and then decreased sharply with increasing Hg concentrations in the media (Fig. 3A). The lowest ABS/CS values were observed in plants treated with 0.4 µM Hg. TR/CS also progressively declined with increasing Hg concentrations (Fig. 3B). The electron transport system (ET/CS) in *L. minor* was highly sensitive to Hg, and complete inhibition of electron transport was observed in plants treated with high Hg concentrations (Fig. 3C). DI/CS continuously decreased with elevating Hg concentrations in the media (Fig. 3D).

### Density of active reaction centers

**Figure 4:**
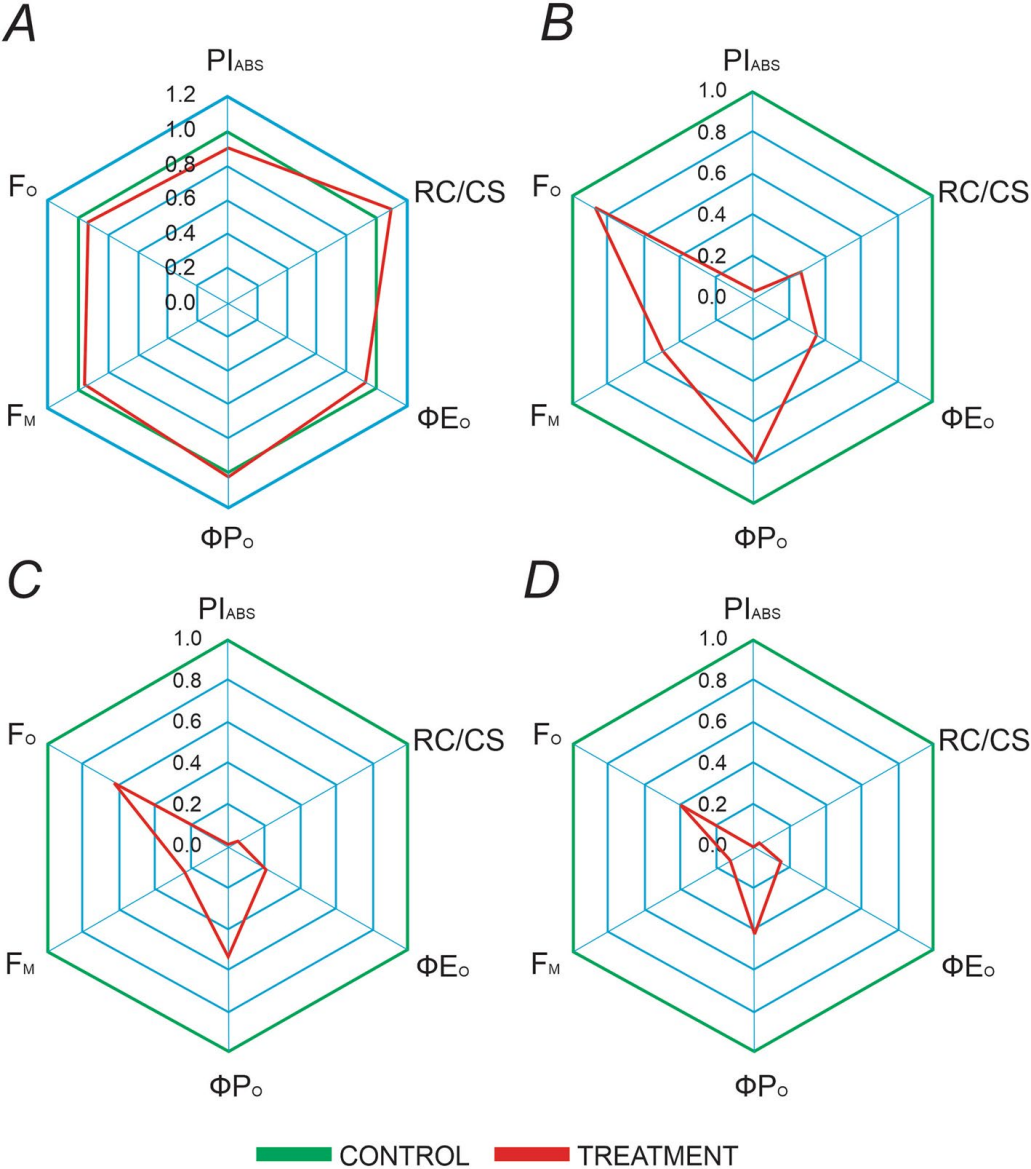
Radar plot of selected JIP parameters (PI_ABS_, performance index; RC/CS, reaction center per cross section; φE_O_, quantum yield for electron transport; φP_O_, maximum quantum yield for primary photochemistry; F_M_, maximum Chl flourescence; F_O_, minimum Chl flourescence) derived from Chl *a* fluorescence of *L. minor* fronds when subjected to various concentration of Hg, (**A**); 0.1 µM, (**B**); 0.2 µM, (**C**); 0.3 µM and (**D**); 0.4 µM. Values presented are the average of three replicates.

The presence of various concentrations of Hg in the growth media induced significant changes in the density of active PSII reaction centers (RC/CS) in *L. minor*. At low Hg concentrations (0.2 µM), a modest increase in active PSII RCs was observed. Conversely, a sharp decline in active RC density was noted as the Hg concentration increased, reaching complete inhibition at 0.4 µM (Fig. 4).

### Yield and flux ratio

The quantum yield of primary photochemistry, represented by F_V_/F_M_ (φPo), was significantly impacted by various levels of Hg in the fronds of *L. minor*. Mild and moderate levels of Hg treatments had little effect on F_V_/F_M_, however, high levels of Hg drastically reduced the quantum yield of primary photochemistry. The lowest value of F_V_/F_M_ was recorded in plants treated with 0.4 µM Hg (Fig. 4). The quantum yield of electron transfer, represented by ET/ABS (φEo), remained relatively unchanged during mild Hg treatment, but significantly declined about 3.5 times with increasing concentration of Hg. (Fig. 4).

### Performance index

The results of the study show that Hg had a pronounced impact on all photosynthetic parameters, including specific energy fluxes, phenomenological energy fluxes, density of reaction centers, and performance indexes, in *L. minor* exposed to various Hg concentrations. The performance index PI_ABS_ was significantly affected by the exposure of Hg, with values reaching zero in the fronds of *L. minor* at high concentrations of 0.2–0.4 µM (Fig. 4). The lowest value of PI_ABS_ was observed in plants subjected to 0.4 µM Hg. The overall effects of Hg-induced stress on all photosynthetic parameters are graphically depicted in the form of a radar plot (Fig. 4).

## Discussion

**Figure 5:**
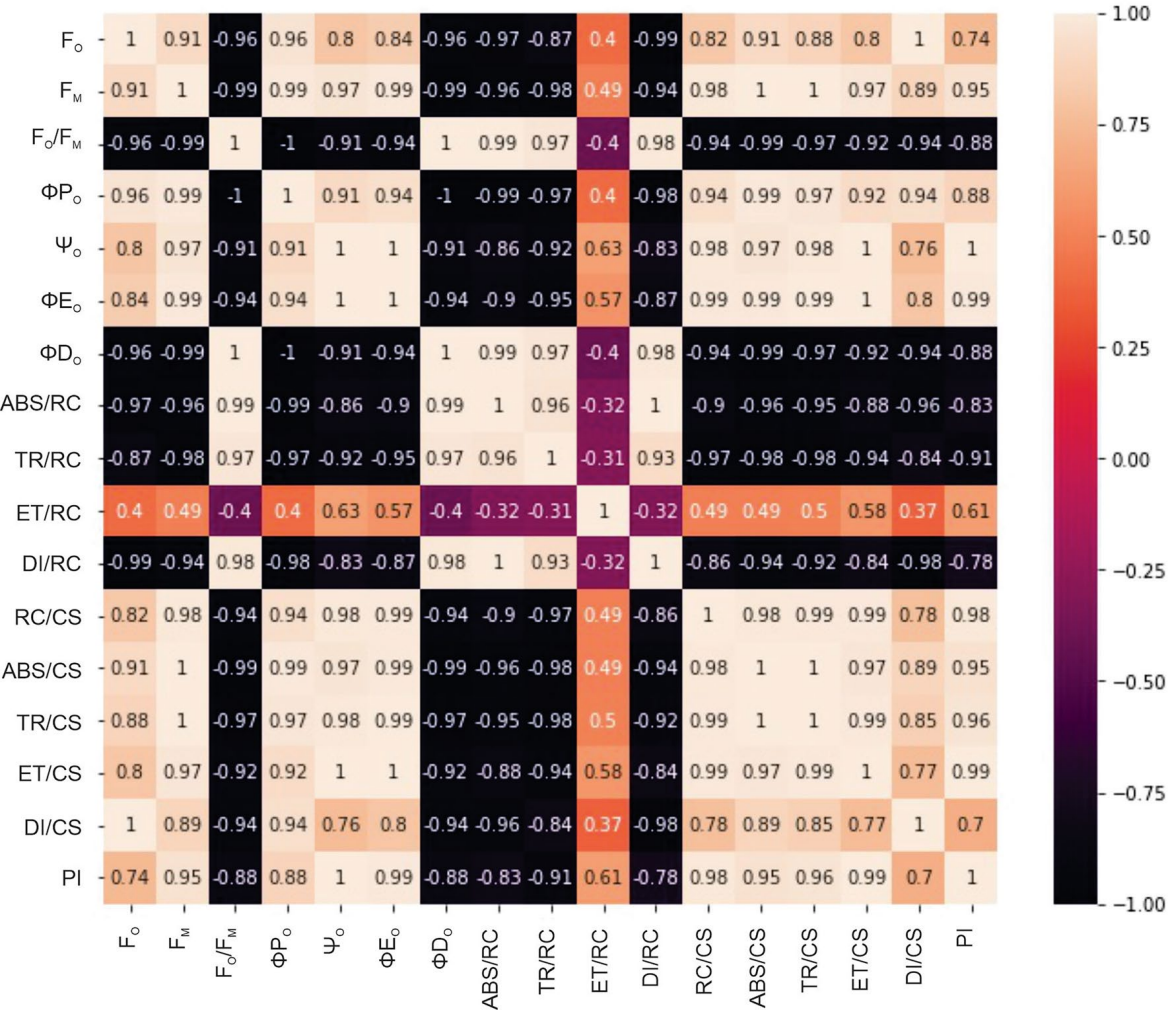
The figure shows heatmap which represents the correlation matrix of the Ch *a* fluorescence parameter of *L. minor* in response to mercury concentrations. The color scale ranges from − 1 (strong negative correlation) to 1 (strong positive correlation), with 0 indicating no correlation. The heatmap was plotted by using google Colaboratory (https://colab.research.google.com/).

The JIP test, which utilizes Chl *a* fluorescence analysis, provides a comprehensive assessment of photosynthetic parameters. This test allows for the evaluation of specific energy fluxes per Q_A_^-^ reducing PSII reaction center (ABS/RC, TR/RC, ET/RC, and DI/RC) and phenomenological energy fluxes per excited cross section (ABS/CS, TR/CS, ET/CS, and DI/CS)^26–28^. The correlation matrix representing the performance of Chl fluorescence parameters was visualized using a heatmap. This heatmap provides a graphical representation of the interrelationships and correlations among the different fluorescence parameters (Fig. 5). Additionally, it enables the determination of the density of active and inactive PSII reaction centers (RC/CS) and various yield or flux ratios, such as φPo and φEo^29,30^. The test also provides an assessment of photosynthetic performance through the calculation of PI_ABS_^16^. The results of the Chl *a* fluorescence analysis in *L. minor* revealed a wide variation in response to different concentrations of Hg. As the intensity of Hg-induced stress increased, there was a continual decrease in the fluorescence signal (F_M_) which suggests the denaturation of the light-harvesting complex (LHC)^26^. The changes observed in the Fo level are linked to the physical interaction of LHC II with the PS II antenna complex, which plays a role in regulating the transfer of energy from the PS II reaction center to other electron acceptor molecules. The decrease in F_O_ and F_M_ values suggests that the treatment with Hg resulted in the degradation of the PS II reaction center^31^.

The presence of a “K” peak in Chl fluorescence transients is widely recognized as an indicator of various types of stress in plants. According to^32^, this peak appears between the O and J peaks, forming the O–K–J–I–P pattern in Chl fluorescence transients and has been observed in various plants subjected to stress conditions such as heat stress^33–35^ and drought stress^36^. However, in a recent study by the authors, it was found that the duckweed species did not exhibit the presence of a “K peak” in their Chl fluorescence curves. Several reports have suggested that the “K step” is related to the inactivation of the OEC^34,36–38^. The absence of a “K peak” in the Chl fluorescence curves of the duckweed species in the present study indicates that the OEC is not negatively impacted by HM treatment. In conclusion, the absence of a “K peak” in the Chl fluorescence curves of the duckweed species studied suggests that the oxygen-evolving complex is not negatively impacted by HM treatment.

The results of the Chl *a* fluorescence analysis indicate that Hg in high concentrations plays a crucial role in disrupting the electron transfer from PSII to PSI. The analysis showed that increased Hg concentration reduced the performance index of PSII (PI_ABS_) and the maximum quantum yield (F_V_/F_M_). The disturbance in electron transfer, caused by Hg treatment, was evident in the JI and IP steps of the OJIP transient curves with increasing Hg concentrations. The results suggest that Hg interferes with light-dependent photosynthesis processes by reducing ATP synthase activity and restricting the flow of protons from the thylakoid lumen to the chloroplast stroma, leading to lumen acidification and a reduction in the oxidation of plastoquinone^39^. Additionally, Hg impacts the photosynthetic apparatus by decreasing the number of active reaction centers (RC) and reducing the rate of electron transfer^40^. The reduction of the rate of the terminal electron acceptors in PSI, as indicated by the I-step of the OJIP transient^41^, was also found to be reduced by Hg treatment.

The growth of *L. minor* with Hg resulted in a significant change in phenomenological energy fluxes per excited cross section compared to control plants. This change is attributed to the inhibition of various plant functions caused by Hg, which forms covalent bonds with the side groups of organic compounds such as proteins, leading to their inactivity^42^. The interaction of Hg with the SH-groups of proteins^43^ is particularly significant and results in the inhibition of enzymes such as protochlorophyllide reductase and plastocyanin, as well as the Calvin cycle enzymes. Additionally, Hg can substitute Mg^2+^ in the Chl molecule. Under Hg treatment, photosynthesis is mainly inhibited at the site of action of lead on the Calvin cycle^44^. The inhibition site is believed to be located at the donor side of PSII, which is less tolerant to Hg than PSI^45^. A 48 h treatment with Hg led to a reduction in the primary quinone acceptor of electrons (Q_A_) in PSII (O–J phase) and a slight change in the quenching of fluorescence controlled by the donor site of PSII and the characteristic activity of the water splitting system (J–I phase) in *L. minor*. The studies revealed that HMs negatively impacted the PSII activity of *L. minor* and the effect varied based on the metal and the time of stress, as the Chl *a* fluorescence parameter, which characterizes PSII activity^46^.

The impact of Hg toxicity on the PSII energy fluxes of *L. minor* was analyzed using energy pipeline leaf models and Biolyzer software. The model depicted the changes in the active and inactive PSII reaction centers per cross-section, as well as the flux of dissipated excitation energy at time zero (DI/CS). The ABS/CS, TR/CS, and ET/CS ratios, which represent the efficiency of light absorption, trapping, and electron transport of PSII, respectively, showed a decline with increasing Hg concentrations^47^. The decrease in ABS/CS indicated a reduction in energy absorbed per excited cross-section, while the decrease in ET/CS indicated lower energy absorption by antenna pigments and inactivation of reaction center complexes^48^. The gradual decrease in the TR/CS ratio demonstrated the significant impact of Hg on the trapping of reaction centers, which could be due to altered absorbance. The observations showed that Hg had a negative effect on PSII by decreasing the density of active reaction centers and increasing the density of inactive centers.

The F_V_/F_M_ ratio is a measure of how effectively primary light energy is converted and captured, and it’s commonly used to assess the quantum yield of primary photochemistry in PSII. If the F_V_/F_M_ ratio declines, it suggests that the presence of Hg is reducing the quantum efficiency of PSII photochemistry either by slowing down the rate of primary charge separation or by disconnecting some minor antennae from PSII^49–52^. The area under the fluorescence induction curve between F_O_ and F_M_ is proportional to the pool size of electron acceptors (Q_A_, Q_B_, PQ, and PSI) on the reducing side of PSII^51^. A reduction in this area in a linear fashion with increasing Hg concentration indicates that electron transfer from the reaction center to the quinone pool is impeded.

In control plants, the Chl *a* fluorescence measurement exhibits a polyphasic rise referred to as the O–J–I–P transient. However, with increasing Hg concentration, there is a significant inhibition of the fluorescence maximum (F_M_) while no change is observed in the minimum fluorescence (F_O_). The inhibition of the J, I, and P phases, which correspond to various stages of electron transport in the photosynthetic process, can occur due to two reasons: first, inhibition of electron transport at the donor side of PSII, leading to the accumulation of P680^+^, and second, due to a decrease in the pool size of the electron acceptor Q_A_^−^^53,54^, which is also reflected by a decrease in the area parameter.

The inhibition of the photosynthetic apparatus by Hg is reflected in the decrease of the ratio of RC/ABS, which represents the density of active PSII reaction centers per Chl^55^. Studies have shown that Hg treatment results in a decrease in the number of active reaction centers per Chl, indicating maximum damage to the water splitting complex of PSII at a concentration of 0.4 μM^56^. This decrease also highlights the disruption of PSII photochemistry^56,57^.

The Performance Index on Absorbance basis (PI_ABS_) is a widely used index for evaluating the primary photochemical reactions of PSII. It incorporates three key structural and functional characteristics of PSII, including the density of active PSII reaction centers per Chl (RC/ABS), the efficiency of light reactions (φ P_O_/(1−φ P_O_)), and the efficiency of dark redox reactions (ψ_O_/(1−ψ_O_)). This index is derived based on the Nernst equation for redox reactions^58^. The results showed a decline in the PI_ABS_ with increasing Hg concentrations (0.1, 0.2, 0.3 and 0.4 μM) as presented in Fig. 4. This decline indicated a negative impact of Hg on the primary photochemical reactions of PSII^57^.

The changes in specific activity parameters of the PSII reaction center are crucial indicators of the absorption and utilization of light energy as well as the reaction center activity^59^. The PSII reaction center is designed to capture light energy for the subsequent transfer of energy. Any remaining energy is dissipated as heat^60^. Our results showed that the ABS/RC and TRo/RC increased significantly in leaves exposed to higher Hg concentrations. This is due to the reduction in the number of active reaction centers per unit area caused by Hg-induced stress, which increases the functional efficiency of the remaining active reaction centers and enhances the specific activity parameters per unit reaction center. This phenomenon is supported by the decrease in the RC/CS values. Furthermore, the increase in the DI/RC value at a Hg concentration of 0.4 μM suggests that the plant activates a self-protective mechanism to reduce excess energy in the PSII reaction center and increase the energy for heat dissipation per unit reaction center.

Based on the present research, it is evident that Hg exposure can induce significant changes in photosynthetic processes in plants, causing inhibition in the photochemical reactions and damaging the PSII reaction centers. This information has important implications for future research on the impacts of HM toxicity on plant growth and productivity. Further studies are needed to understand the underlying mechanisms of Hg toxicity on photosynthesis and to develop effective strategies for mitigating the negative effects of Hg on plant growth and development. Additionally, this information can be useful for environmental monitoring programs and for risk assessment of HM pollution in agricultural and natural ecosystems. By better understanding the impacts of Hg on photosynthesis, it is possible to develop effective measures to protect the health of plants and ensure the sustainability of food production systems.

## Materials and methods

### Plant materials, growth condition and HM treatment

The present study followed the OECD, 2002 guideline (with slight modifications) for testing of chemicals^61^*L. minor* plants were collected from a freshwater pond located at 24°35′56.12″ N, 73°42′17.79″ E in Udaipur City, India. The fronds were acclimatized in a growth medium (10% Hoagland’s) under laboratory conditions with a light irradiance of 140–150 μmol/m^2^/s, a 14-h photoperiod, and a day/night temperature of 25/20 °C. Pre-cleaned round-shaped glass bottle of 500 ml capacity were used, and five concentrations of 0.0, 0.1, 0.2, 0.3, and 0.4 μM Hg were prepared from the stock solution. All concentrations were prepared in nutrient medium. Fresh biomass of 30 g of *L. minor* was taken out from culturing tank and treated with different concentrations of Hg metal in 300 ml media. After 48 h, Chl *a* fluorescence analysis was performed to obtain various parameters, including F_O_, F_M_, Fo/F_M_, φPo, φEo, ABS/RC, TRo/RC, ETo/RC, DIo/RC, RC/CSm, ABS/CS, TRo/CS, ETo/CS, DIo/CS and PI_ABS_.

### Measurement of polyphasic chlorophyll fluorescence kinetic

In the experiment, measurements were performed on dark-adapted *Lemna* fronds using the Handy PEA instrument (by Hansatech Instruments Ltd., located in Norfolk, UK) after a minimum of 30 min of dark adaptation. Fluorescence rise OJIP curves were induced through a 1-s pulse of red light (650 nm, 3500 μmol/m^2^ s). The fluorescence transients were recorded over a leaf area of 4 mm diameter using a red-light source with a peak at 650 nm and an intensity of 3000 µmol/m^2^ s, which was sufficient to close all PSII reaction centers and obtain a true F_M_ fluorescence intensity. This was achieved through the use of a high-intensity LED array consisting of three light-emitting diodes. A measuring time of one second was consistently utilized throughout the experiment. Following primary fluorescence data were obtained from OJIP test:

*F*_*O*_ = *F*_*50 ms*_*, fluorescence intensity at 50 ms*

*F*_*150*_ = *Fluorescence intensity at 150 ms*

*F*_*300*_ = *Fluorescence intensity at 300 ms*

*F*_*J*_ = *Fluorescence intensity at the J-step (at 2 ms)*

*F*_*M*_ = *Maximal fluorescence intensity*

*t*_*Fmax*_ = *Time to reach F*_*M*,_* in ms*

*Area* = *Area between fluorescence curve and F*_*M*_

### Specific energy fluxes or specific activities

The specific energy fluxes (per Q_A_-reducing PSII reaction center-RC) such as ABS/RC (absorption flux per RC), TR/RC (trapped energy flux per RC), ET/RC (electron transport flux per RC), DI/RC (dissipated energy flux per RC) were calculated by following equations^62^.

$$ ABS/RC = M_{O } \times \left( {1 - V_{J } } \right) \times \left( {1/\varphi P_{O } } \right) $$

$$ TR/RC = M_{O } \times \left( {1 - V_{J } } \right) $$

$$ ET/RC = M_{O } \times \left( {1 - V_{J } } \right) \times {\Psi }_{O } $$

$$ DI/RC = \left( {ABS/RC} \right) - \left( {TR_{O } /RC} \right) $$

where Ψ_O_ = 1−V.

### Phenomenological energy fluxes or phenomenological activities

The phenomenological energy fluxes (per excited cross section-CS) such as ABS/CS (absorption flux per cross section), TR/CS (trapped energy flux per cross section), ET/CS (electron transport flux per cross section) and DI/CS (dissipated energy flux per cross section) were calculated by following equations^62^.

$$ ABS/CS = F_{O} \;or\; other\; usefull\; expression $$

$$ TR/CS = \varphi P_{O} \cdot \left( {ABS/CS} \right) $$

$$ ET/CS = \varphi P_{O} \cdot {\Psi }_{O} \cdot \left( {ABS/CS} \right) $$

$$ DI/CS = \left( {AB/CS} \right) - \left( {TR/CS} \right) $$

### Density of active PSII reaction centers (RC/CS)

The concentration of active photosystem II (RC/CS) was measured by using the following equation of JIP test^62^.

$$ RC/CS = \varphi P_{o} \cdot \left( {V_{J} /M_{0} } \right) \cdot F_{M} $$

### Quantum efficiencies or flux ratios

φP_O_ (maximum quantum yield of primary photochemistry) and φE_O_ (quantum yield of electron transport) were calculated by following equations^62^.

$$ \varphi P_{O} \, or \,  TR/ABS = 1 - \left( {F_{O} /F_{M} } \right)  \, or \,  F_{V} /F_{M} $$

$$ \varphi E_{O}  \, or \,  ET/ABS = [1 - \left( {F_{O} /F_{M} } \right) \cdot {\Psi }o $$

### Performance index

Photosynthetic performance index PI_ABS_ (performance index on absorption basis)was calculated by the following equation:

$$ PI_{ABS} = \frac{{1 - \left( {F_{O} /F_{M} } \right)}}{{M_{O} /V_{J} }} \times \frac{{F_{M} /F_{O} }}{{F_{O} }} \times \frac{{1 - V_{J} }}{{V_{J} }} $$

### Heatmap

To further explore the relationship between the parameters, a heatmap was created using the Seaborn library. The heatmap represented the correlation matrix of the parameters and provided a visual representation of the strength and direction of the relationships between the parameters. The color scale of the heatmap ranged from − 1 (strong negative correlation) to 1 (strong positive correlation), with 0 indicating no correlation. The heatmap was created using the sns.heatmap function and the correlation matrix was passed as a parameter. The heatmap allowed for a quick visual assessment of the relationships between all of the parameters, highlighting any strong correlations and providing a more comprehensive view of the data compared to the regression plots. In conclusion, the regression plots and heatmap provided a visual representation of the relationships between the chlorophyll *a* fluorescence parameter of *L. minor* in response to Hg concentration. The results showed the relationship between the parameters and provided a deeper understanding of the effect of Hg on the chlorophyll *a* fluorescence of *L. minor*.

### Statistical analysis

The statistical analysis was performed using one-way analysis of variance (ANOVA) and the Tukey HSD test with a significance level of p = 0.05 using the SPSS (22.0) software by IBM (Armonk, NY, USA). Only the results with statistical significance (p ≤ 0.05) are displayed in the figures.

## Conclusion

The present investigation demonstrated that the increasing concentration of Hg had a negative impact on *L. minor*. Chlorophyll fluorescence analyses suggested that the photosynthesis process was primarily affected under Hg treatments. Hg treatment may hinder the functionality of PS II and ATP synthesis, which ultimately led to negative effects on duckweed survival. Hg exposure affected phenomenological energy fluxes per excited cross-section and specific activity parameters of the PSII reaction center. Energy absorption, trapping efficiency, and energy dissipation were all affected, which can be attributed to Hg’s interaction with proteins and inhibition of key enzymes involved in photosynthesis. Furthermore, high concentrations of Hg disrupted electron transfer from PSII to PSI, resulting in reduced PI_ABS_ and F_V_/F_M_ of PSII. The OJIP parameters analyzed in this paper have the potential to serve as an effective tool for quickly identifying the main way in which harmful substances affect the photosynthesis in plants.

## Data Availability

The data and materials that support the findings of this study are available from the corresponding author upon request.
